# Lessons From a Behavior Change Intervention to Improve Provider-Parent Partnerships and Care for Hospitalized Newborns and Young Children in Kenya

**DOI:** 10.9745/GHSP-D-23-00004

**Published:** 2023-11-30

**Authors:** Charlotte E. Warren, Pooja Sripad, Charity Ndwiga, Chantalle Okondo, Felicitas M. Okwako, Caroline W. Mwangi, Timothy Abuya

**Affiliations:** a Population Council, Washington, DC, USA.; b Population Council, Nairobi, Kenya, USA.; c Bungoma County Referral Hospital, Bungoma, Kenya.; d Division of Newborn and Child Health, Ministry of Health, Nairobi, Kenya.

## Abstract

Enhancing respectful, responsive, integrative, and nurturing care for hospitalized newborns and young children (aged 0–24 months) is globally recognized but under-researched in low- and middle-income countries. Responsive, family-centered interventions target providers and parents and emphasize partnership in caring roles. From February 2020 to August 2021, we engaged in a participatory co-creation process with parents, providers, and newborn and child health stakeholders in Kenya to develop a comprehensive provider behavior change intervention and implemented it across 5 hospitals in Nairobi and Bungoma counties in Kenya. The multifaceted intervention included a 7-module orientation, feedback meetings, job aids, and psychosocial support—leveraging in-person and remote modalities—for providers working in newborn and pediatric units. We used a mixed-methods evaluation drawing on a pre-post provider survey, pre-post qualitative interviews with providers and parents, and a follow-up parental survey. There were significant post-intervention improvements in provider knowledge on safeguarding sleep, positioning and handling, and protecting skin. However, there were also significant reductions in providers' knowledge in identifying a child's pain, parental stress, and environmental stress. Among parents who received coaching from providers, there were higher levels of interpersonal communication between parent and provider, parental empowerment, and improved ability to provide integrated, responsive care to their child. Despite the challenges of implementing a provider-focused intervention to improve care for hospitalized newborns and young children during the global COVID-19 pandemic, we have demonstrated that it is feasible to implement a hybrid virtual and in-person process to influence several outcomes, including provider knowledge and practice, improved provider partnerships with parents, and parents' capacity to engage in the care of their newborn or young child.

## Abstract (precis)

Strengthening provider-parent partnerships through improved communication enhances the respectful, responsive quality of newborn and young child care, which is critical to positive health outcomes.

## Teaser

**Key Findings:**

- We developed a provider behavior change intervention to improve communication between providers and parents of hospitalized newborns and young children aged up to 24 months that focused on orientation and emotional support for providers, coaching and emotional support for parents, and monitoring for structural change.
- The intervention influenced outcomes including having a positive association in improving providers' knowledge of respectful, responsive nurturing care, improving provider-parent communication and partnership, improving the ability of parents to provide responsive care to their newborns, and increasing parent empowerment.
- The providers' understanding of parents' needs and parents' understanding of providers' working environment resulted in a pragmatic behavior change intervention promoting responsive care of hospitalized newborns and young children.

**Key Implication:**

- Researchers and program managers should consider our experience as a successful example of how to apply a nurturing, integrative, and responsive approach to the care of hospitalized newborns and young children in low-resource settings.

## INTRODUCTION

Despite the worldwide progress in reducing child mortality over the past few decades, a child's ability to survive and thrive remains an urgent concern. Global guidelines recognize the critical roles of parents, caregivers, and families in promoting the well-being and development of newborns and young children,^1^ including their experiences when hospitalized.^2^^–^^4^ One recognized approach to supporting children to achieve their full potential for physical, social, emotional, and cognitive development is the World Health Organization (WHO) Nurturing Care Framework.^1^^–^^3^ The framework promotes good health, adequate nutrition, safety and security, responsive caregiving, and opportunities for learning among all children.^1^ Additional global frameworks guide the care of hospitalized newborns and very young children,^2^^,^^3^^,^^5^ as well as approaches such as the Neonatal Integrative Developmental Model that includes optimizing nutrition, minimizing pain and stress, positioning and handling, safeguarding sleep, and protecting the skin.^4^ However, there are challenges to the uptake and effects of these approaches on hospitalized newborns and young children in low-resource settings.

Enhancing respectful and responsive care of hospitalized newborns and young children (aged 0–24 months) is critical to promoting positive health and developmental outcomes, yet few efforts have been made to define or measure the quality of care and experience of these groups and their caregivers, especially in low- and middle-income countries.^6^ Because young children are unable to voice their experiences, there is a need to understand and address facility-based experiences of parents (both mothers and fathers) with hospitalized newborns and young children aged up to 24 months.

There is a need to understand and address facility-based experiences of parents with hospitalized newborns and young children to improve care and promote positive health and developmental outcomes.

Literature shows that positive and negative experiences of care are related to a range of individual, social, and normative factors constituting a “health systems culture” that affect how providers interact with parents and caregivers.^7^ Lack of autonomy in making choices on treatment and medical procedures for parents of very young children is associated with maternal post-traumatic stress and anxiety.^2^^,^^7^ Long periods of separation between mother and baby, especially in the neonatal intensive care unit, stress both mother and newborn and hinder bonding, with potential long-term consequences for the mother-baby relationship.^3^^,^^4^ Overdiagnosis of risk conditions in very young children and inappropriate use of drugs, often with unnecessary intravenous or intramuscular treatments, are also perceived as negative experiences by parents of hospitalized young children.^8^

Other negative care experiences are associated with normalized health system practices and structures, as well as individual provider attitudes and behaviors. Health system factors, such as weak implementation of official guidance and infrastructural limitations, outdated “rules” around visiting times that prevent working fathers from seeing their sick child, and strict feeding times for very young children, contribute to the mistreatment, poor quality of care, and neglect of hospitalized young children. Lack of communication between providers and parents is often ascribed to limited staffing and provider workloads.^8^

Provider training and knowledge, effective communication, parental counseling by providers, and supply and equipment availability affect the efficacy of evidence-based practices, including integrated management of newborn and child illness, nurturing care, integrative developmental care, kangaroo mother care, and early and exclusive breastfeeding.^4^^,^^9^ The first 1,000 days (from pregnancy to 24 months) is the foundation of lifelong learning and development.^10^ There is a compelling case to focus on fundamental elements, including interpersonal communication and interactions between providers and parents and caregivers, provider norms and attitudes toward parental involvement (both mothers and fathers), psychosocial elements including the need for emotional support, and the need for feedback loops for parents and caregivers and providers.

In Kenya, the Ministry of Health (MOH) adopted the WHO Nurturing Care Framework, using a multisectoral approach where the focus is on providing high-quality essential health services from birth throughout childhood in a responsive manner, including several policies seeking to improve early childhood development at the household, community, and health facility levels. This includes nutrition, sleep, skincare, play, cognitive development, parents' involvement, and counseling. A new policy document that is not yet official, the Newborn and Child Health Strategic Plan (2022–2026), has also integrated early childhood development. Although there is a robust policy framework originating from central government, counties are responsible for “domesticating” any policies to fit their individual contexts and are responsible for designing, budgeting, and introducing new services. Implementation of these policies at the health facility level has been uneven, with reliance on implementing and development partners and other stakeholders to support pilot studies and broader interventions.^10^

In this study, we sought to evaluate a provider behavior change (PBC) intervention implemented in 5 hospitals in Nairobi and Bungoma counties in collaboration with the Kenya MOH (national and county levels) and its effect on provider knowledge and practice related to respectful, responsive care; teamwork/peer support; communication between providers and parents; and parents' capacity to engage in the care of their newborn or young child while in the hospital.

## METHODS

### Study Setting

The study was co-implemented by Population Council and the Newborn and Child Health Division at the MOH, and the county governments of Bungoma and Nairobi, in 5 tertiary hospitals in Bungoma and Nairobi in Kenya. Bungoma County, located in Western Kenya, represents a rural area with a total fertility rate of 5.0, which is higher than the national average of 3.9. In contrast, Nairobi County, which also serves as the capital, represents an urban area with a total fertility rate of 2.7. Both counties have neonatal (aged 0–4 weeks) mortality rates higher than the national average of 22 per 1,000 live births: 33 per 1,000 live births in Bungoma and 39 per 1,000 live births in Nairobi, which is also the highest in Kenya.^11^ Infant (aged younger than 12 months) mortality rates in the 2 counties are also higher than the national estimates of 39 per 1,000 live births: Bungoma 97 per 1,000 live births and Nairobi 60 per 1,000 live births.^11^ In Bungoma County, data were collected from 2 public referral hospitals; in Nairobi County, data were collected from 3 hospitals: 1 large public maternity hospital, 1 large tertiary public hospital, and 1 faith-based hospital. Study facilities were selected in consultation with the county health management teams and the national MOH. The study population involves providers working in newborn and pediatric units and parents and caregivers of hospitalized newborns and young children aged 0–24 months.

**FIGURE 1:**
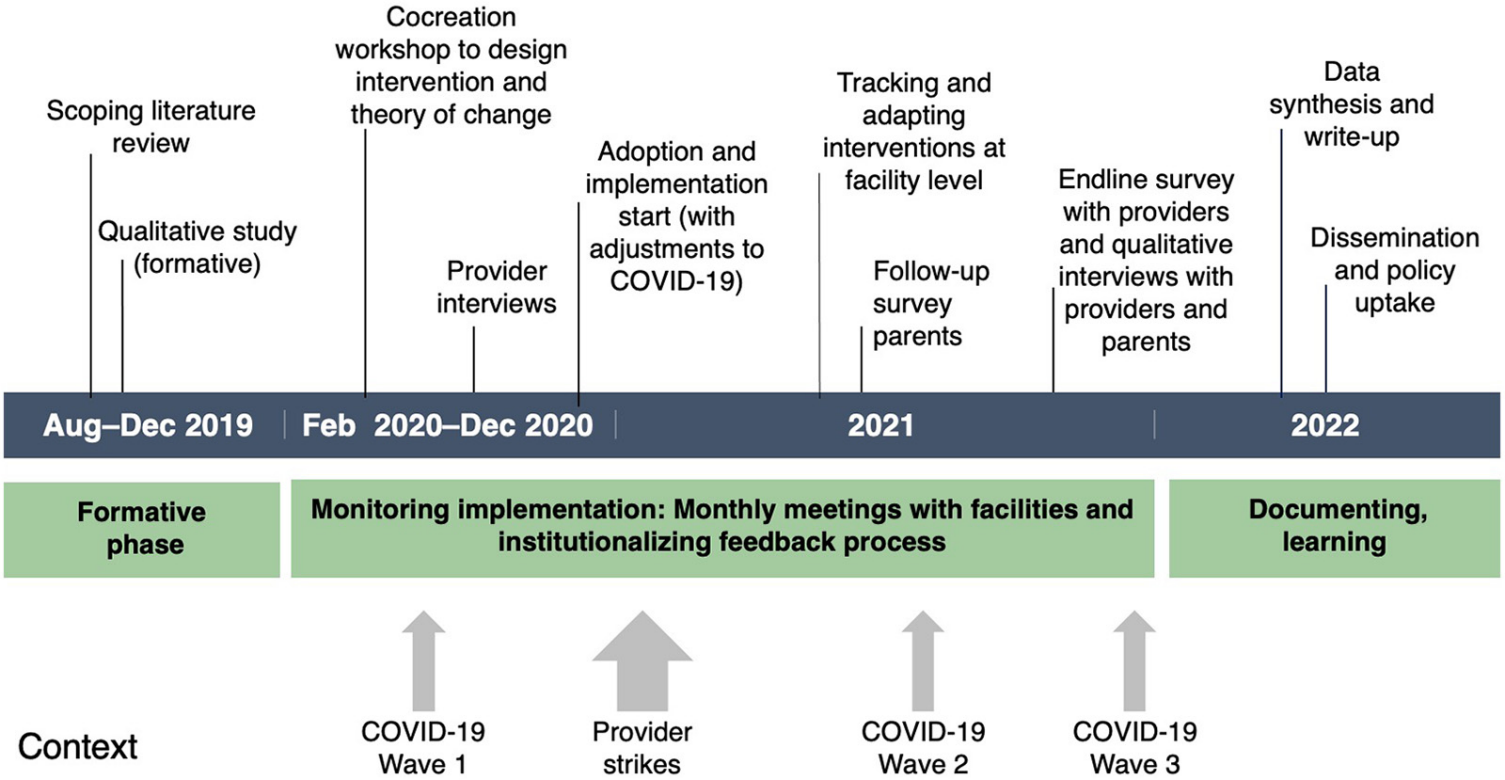
Implementation Research Approach Timeline for Provider Behavior Change Intervention in Kenya

We conducted a mixed-methods evaluation of a multifaceted intervention aimed at improving the knowledge, practice, communication skills, and emotional needs of providers working with hospitalized newborns and young children, as well as the experiences of hospitalized newborns and young children and the emotional needs of their parents across 5 study hospitals. Using an iterative implementation research approach, we drew on several data sources, including a pre-post provider survey, follow-up parental survey during implementation, periodic qualitative in-depth interviews (IDIs) with providers and parents, pre-intervention focus group discussions (FGDs) with parents, and monitoring data (Figure 1).

### Intervention Design

After a formative study conducted in 2019, we engaged in a participatory cocreation process to draft a theory of change and develop and implement the intervention. The cocreation process (early February 2020) included parents of recently hospitalized newborns and young children (aged 0–24 months), health care providers (nurses, midwives, clinical officers, and doctors) working in maternity, newborn, and pediatric units in the 5 study hospitals, MOH policymakers, and other newborn and child health stakeholders in Kenya. We invited parents (with newborns or young children who had been hospitalized in the last few months) and providers who had taken part in the formative study to a 2-day residential meeting. The formative findings were shared in plenary, after which separate discussions were held for mothers, fathers, and providers. Cots were provided in the meeting rooms, and breastfeeding breaks were incorporated into the day. There were sufficient facilitators to help hold the babies (if awake and settled and with parents' permission) to enable parents to fully participate in the discussions.

A second workshop was held with newborn and child health stakeholders, where the formative data and outcomes from the first workshop were reviewed and discussed, including the contextual challenges of implementing existing newborn and young child policies in Kenya at the time. The intervention incorporated elements from a range of theories and concepts around newborn and infant neurodevelopment, nurturing and responsive care, and parental involvement in a healing environment (Box 1)^2^^,^^4^^,^^12^ and drew from a literature review of experience of care of hospitalized young children.^13^

BOX 1 Frameworks, Models, and Approaches for Newborn and Young Child Health Care Reviewed and Adapted to Develop the Theory of Change^1^^–^^5^^,a^

- The Nurturing Care Framework (2018): Includes good health, adequate nutrition, safety and security, responsive caregiving, and opportunities for early learning.
- World Health Organization Quality of Maternal and Newborn Health Care (2016): Experience of care includes 3 domains: (1) effective communication, (2) respect and preservation of dignity, and (3) emotional support.
- World Health Organization Quality of Child and Young Adolescent Health Care (2018): Experience of care includes 3 domains: (1) effective communication and meaningful participation; (2) respect, preservation of dignity, protection, and fulfillment of child rights; and (3) emotional and psychological support.
- Neonatal Integrative Developmental Care Model (2016): Includes partnering with families, optimizing nutrition, minimizing pain and stress, positioning and handling, safeguarding sleep, and protecting skin within a healing environment.
- Family-Centered Developmental Care (2018): A framework of newborn care that incorporates theories and concepts of neurodevelopment, neurobehavior, parent-infant interaction, parental involvement, breastfeeding promotion, environmental adaptation, and change of hospital systems.

^a^See Figure 1.

The implementation approach also emphasized aspects of the Research and Learning Agenda for Advancing Provider Behavior Change Programming developed by Breakthrough RESEARCH that focuses on addressing norms and conditions that shape provider behaviors and parent experience.^14^ Extant literature and the cocreation process identified that provider behavior and the ability to provide quality care are affected by work-related stress and burnout due to heavy workload, poor working conditions, and a lack of supportive supervision and psychosocial support.^15^^–^^17^

Although the cocreation process included 2 in-person workshops, implementation of the intervention took place after the onset of the COVID-19 pandemic. The original design was adapted to enable virtual capacity-building using a mentoring approach, including interactive virtual “jam board” sessions followed by in-person activities (i.e., teams already working together at the facility level) to heed MOH restrictions, prevent spread, and protect providers and patients.

We developed a multifaceted intervention that focused on understanding providers' personal values, building knowledge and skills on communication, integrative and nurturing care, and emotional support and partnerships between providers and parents. We also developed/adapted the following 4 key job aids to assist providers and parents (Supplement 1).

- “Communication During Hospitalization”: a charter for providers and parents focused on admission, care plans, ward rounds, feeding support, insertion of a nasogastric tube or intravenous infusion, and the ward environment.
- “Parents' Emotional Wellness: Reduce Distress, Emotional Support, and Partnership (DEP) Guide for Supporting Parents During a Young Child's Illness”: a resource to help identify sources of anxiety, fears, and concerns; provide emotional support; and engage with parents and families to mitigate stress.
- “Providers' Emotional Awareness”: the A, B, Cs (awareness, balance, connection) of provider self-care when working with sick children and their families.
- “What Can Men Do?”: information for fathers and other male caregivers on what they can do for their newborns and young children.

The intervention focused on understanding provider personal values, building knowledge and skills on communication, integrative and nurturing care, and emotional support and partnerships between providers and parents.

One component of the intervention to support providers in improving their interactions among themselves and with parents included a 7-module orientation on: (1) promotion of awareness and practice of responsive and nurturing care, (2) communication skills, (3) how to coach parents of hospitalized young children, and (4) how to build provider capacity for emotional support, including enhancing provider mental health and subsequent parental experience. The orientation package was developed by Population Council and 5 cofacilitators, a county pediatrician, and members of the County Child Health Division, National Newborn and Child Health Division, and MOH. Nurses and midwives from the newborn/pediatric units from the 5 hospitals who had experience in mentoring or expressed an interest in mentoring comprised the 21 mentors who attended a 5-day mentors' training workshop (virtual). The final selection was done jointly by the hospital management and newborn unit (NBU) and pediatric ward providers. This was followed by mentor-led continuous professional development sessions and individual on-the-job training to build provider knowledge, capacity, and skills.

Capacity-building of providers focused on (1) provision of high-quality respectful care, interpersonal communication, and interactions with parents including fathers and (2) facilitation of better parent-provider engagement through increased awareness and coaching around essential integrative care elements (optimizing nutrition, protecting the skin, minimizing stress and pain, safeguarding sleep, and positioning and handling) and early childhood physical and cognitive development. Mentors led facility-based orientation sessions targeting a cohort of providers, used combinations of standardized slide decks, discussed print and video-based job aids, and engaged in individual mentoring/on-the-job training. Role-play also included pictures where providers used dolls to demonstrate to their colleagues how to handle babies and how to teach parents to play and interact with their child. Another key component of the intervention was regular virtual feedback meetings held with providers from the 5 study sites to discuss progress and provider group and individual psychosocial support sessions at each hospital.

Providers drew on a range of materials to routinely coach parents during a child's hospitalization. These materials were primarily from the Kenya MOH Early Childhood Development training package but also included those developed for the intervention or adapted from global or regionally applied tools (Supplement 1). Providers coached parents on integrative developmental care and nurturing care and encouraged them to ask questions and voice their opinions or concerns for shared decision-making about their young child's care while in the hospital. Each facility displayed job aids, including adapted wall charts and protocols for young childcare, a provider-parent communication charter, and targeted messaging and posters on how fathers can participate within pediatric wards, NBUs, and waiting areas (as previously described). For example, parents were instructed on nasogastric tube feeding, breastfeeding, ways to make their child comfortable, skincare, and play for cognitive development. The intervention components of focus in this article include orientation and emotional support for providers and the subsequent transfer of knowledge and support to parents vis-à-vis coaching interactions.

### Data Sources

We triangulated qualitative and quantitative information collected from intervention participants—targeted providers and beneficiary parents—to describe changes in experiences of provider-parent partnership and engagement as they translate to parents' empowerment to provide responsive care for their hospitalized child. We drew on mixed data sources to garner lessons about the multifaceted intervention's effectiveness, implementation process/experience, and perceived changes across multiple perspectives. Pre- and post-quantitative surveys with providers were conducted with purposively sampled health care providers working in maternity, postnatal, newborn, and pediatric wards before (n=154) and after (n=104) the intervention was implemented 12 months apart. Provider surveys explored levels of knowledge and capacity around integrative responsive nurturing care, respect, interpersonal communication, and reflections on the provider's interactions with parents (Figure 1).

A follow-up survey of parents of newborns and young children (n=382) who were hospitalized in either the NBU or the pediatric wards was conducted toward the end of the intervention implementation (months 8–9), exploring their experience of care, interpersonal communication with providers, empowerment, and ability to responsively care for their child, including awareness and engagement on nurturing care elements. The sample of parents was based on those who had responded to a short exit survey at the time of their child's discharge from the study hospital that asked them a few questions and granted permission to be called by a study team member at a future date for a longer follow-up survey about their experience during their child's hospitalization.

In-depth interviews (IDIs) with providers (n=16) from the 5 hospitals elicited how the intervention components affected their ability to coach and interact with parents. IDIs with a sample of parents from all 5 hospitals before the intervention (n=25) and a sample of parents after the intervention (n=17) from the 5 hospitals describe the change in their experience communicating and interacting with providers during their child's hospitalization following the implementation. Both providers and parents were purposively sampled—the former based on their experience undergoing trainings and/or engaging with parents within the intervention components (all mentors that led implementation in their respective facilities were interviewed qualitatively) and the latter based on their experience having a newborn or young child that was hospitalized. Pre-intervention, the research team initially visited the 5 hospitals to introduce the study to managers and department heads. Parents were approached in person by the research team with assistance from hospital staff. We also used patient records to identify parents with children in the target age group (0–24 months), and hospital staff introduced the research assistants to the parents. Post-intervention, ward in-charges compiled a list of parents that had been discharged and gave permission to be interviewed; facility records and community health volunteers were then used to trace the parents.

Research assistants were trained on how to build rapport and trust with parents in person and over the phone, including being compassionate and having regular attention checks to see if the respondent was willing to continue with the interviews. They stopped any interviews if parents became distressed and referred cases for counseling or psychosocial support where needed. All in-person interviews were conducted in a private room in the facility away from providers. Phone interview respondents were encouraged to take the phone call in a private room in their homes for confidentiality purposes.

Pre-intervention FGDs (n=12) were conducted with men and women whose child had recently been hospitalized in any of the 5 study hospitals. FGDs explored normative views of men and women around their needs while engaging and caring for their hospitalized children as well as their interactions with health providers. Further details for pre-intervention qualitative data collection, sampling, and structure can be found elsewhere.^18^

Monitoring data in the form of provider peer feedback forms and process documentation were also included to help further contextualize the findings and speak to elements of change in provider behavior, particularly those that related to their experience of intervention elements and interactions with parents.

All study participants (providers and parents) gave their consent. They were informed about the aim of the study and its methods, institutional affiliations of the research, information on the process of participation, anticipated benefits, potential risks, follow-up, duration, compensation, potential discomfort, the right to abstain or to withdraw at any time without reprisal, and measures to ensure privacy and confidentiality of all information provided.

### Data Collection

All quantitative and qualitative data were collected by 35 trained research assistants with at least a bachelor's degree in the social sciences or public health or practicing clinicians (clinical officers and nurses) working in other facilities with some experience in similar study techniques. Study researchers trained data collectors on the study purpose and methods, reviewed research ethical protocols, and provided opportunities to practice/role-play interview-setting scenarios. They also supported data collectors throughout fieldwork with daily debriefing and target assessments via WhatsApp chats, ad hoc support in responding to any questions the team had, and a final debriefing session. Other than the pre-intervention qualitative data collected in person, the remaining data were all phone based, given prevailing COVID-19 restrictions.

Structured quantitative surveys lasted around 1 hour; responses were collected using Open Data Kit software and analyzed using Stata 16. The provider survey also included a self-administered section, where providers were sent a link and participant ID to complete the survey. Open-ended qualitative data collection sessions took 60–90 minutes. Data were collected in English or Kiswahili using structured interview guides, audio-recorded, transcribed, translated into English, and anonymized. Translations were done simultaneously with transcription. Research assistants who conducted the IDIs or FGDs would translate the audio-recordings from Kiswahili into English. All completed transcriptions were quality checked (by the team leader and/or peers) against the audio to correct any errors and ensure the meaning remained. In some instances, Kiswahili phrases were left in quotations if the study team felt any meaning was lost in translation. Qualitative data were analyzed using NVivo 12. Additionally, monitoring data collected from providers using a simple self-administered electronic tool hosted on a KOBO platform (a free toolkit used for collecting and managing data in challenging environments) sent to providers monthly over 3 months was used to assess peer-observed interactions with parents on various elements of nurturing and respectful care. The project held monthly meetings with providers where we reviewed the monitoring data and discussed areas of success and improvement. Upon completion of the assessment, data would be received immediately on a project portal and analyzed; we selected to use these data instead of the self-reported data in the provider survey, which may have shown bias to overreporting.

### Analysis

We drew on several key quantitative measures in our analysis (Box 2). At the provider level, key measures included pre-post knowledge levels on respectful and responsive care for newborns and young children, each representing a range of index scores. These index scores, summarized in Box 2 and detailed in Supplement 2 Table S1, covered the 5 key elements of care identified for the intervention. At the parent level, we used a proxy composite indicator (15 items) for intervention exposure that speaks to having “received information on childcare through intervention.” We explored 3 primary outcomes comprising composite measures of parents rating the quality of (1) interpersonal communication shown by providers in their interactions during their child's hospitalization (16-item index), (2) parent empowerment in caring for their child (7-item index adapted from the validated scale in McClair et al.^19^), and (3) parents' self-reported ability to provide integrative responsive care (27-item index for newborns aged 0–28 days and a 21-item index for very young children aged 29 days to 24 months). Bivariate chi-squared tests assessed pre-post changes in providers' nurturing care knowledge, while adjusted linear regressions assessed the associations between parent exposure to the intervention and the 3 self-reported outcomes (interpersonal communication, empowerment, and integrative responsive care ability). Stata 16 was used for quantitative analysis.

BOX 2 Summary Measures Used at Provider and Parent Level to Evaluate the Provider Behavior Change Intervention

**Provider Survey**

To assess providers' knowledge on 5 key elements of integrative responsive care, the provider survey asked several questions depending on the age of the child, including items that cover how to coach parents to engage in their child's care (Supplement 2 Table S1).

- Minimizing stress and pain
  - 7 items for a child's pain and stress
  - 5 items for parental stress
  - 5 items for environmental stress
- Optimizing nutrition
  - 14 items for newborns
- Safeguarding sleep
  - 12 items for newborns
  - 11 items for older children
- Positioning and handling
  - 8 items for newborns
  - 8 items for older children
- Protecting skin
  - 10 items for newborns
  - 7 items for older children

**Parent Follow-Up Survey**

- To assess parents' experience with the intervention, the parent follow-up survey asked a series of questions on what information they received during hospitalization. This was a proxy composite indicator for intervention exposure (15-item index) (Supplement 2 Table S2).
- To assess the quality of interpersonal communication between parents and providers, parents were asked to rate the quality of communication shown by providers in their interactions during their child hospitalization (16-item index) (Supplement 2 Table S3).
- To assess whether parents were empowered to care for their children during hospitalization, an empowerment scale (7-item index) was adapted from the validated scale by McClair et al. (Supplement 2 Table S4).^19^
- To assess parent's knowledge and ability to provide the 5 elements of integrative responsive care, the survey asked parents questions depending on the child's age (Supplement 2 Table S5).
  - 27 items for newborns
  - 20 items for older children

Qualitative transcripts were reviewed by a team of experienced local researchers in the respectful maternity care and quality-of-care space to derive a codebook that captured themes that emerged inductively and deductively based on our study questions related to the intervention. These researchers had advanced public health and/or social science degrees, understood the local norms and language, and spent extensive time in conversations with both clients and health providers working in hospital settings. They also participated in the cocreation meetings and multiple phases of data collection, which enabled them to bring a deep understanding of respondent perspective and context to the analysis. A team of 3 researchers applied the codebook to all the data pre- and post-intervention, followed by a charting process led by the study team. Reflexivity was enhanced through intermittent discussions on what the results were illustrating to reduce personal biases in interpretation. Memos were developed to describe provider and parent perspectives of the intervention, including how the various tools and materials were useful in providing and receiving care, as well as descriptions of any changes experienced from varied provider and parent perspectives. Nvivo was used for analysis.^2^

Triangulating across data sources, we present our results in 4 broad themes around the study aims, with a focus on effects and mechanisms affecting provider-parent partnership and engagement around respectful responsive care: (1) building of provider knowledge and capacity for integrative responsive care; (2) quality of provider-parent communication; (3) provider-parent coaching empowering parents in integrative responsive care; and (4) associations between intervention exposure and parents' self-reported outcomes.

### Ethical Approval

This study was approved by the Institutional Review Board at the Population Council. The study was also reviewed and approved by the AMREF Ethics and Scientific Review Committee and licensed to conduct the research by the National Commission for Science Technology and Innovation (NACOSTI/P/20/6478).

## RESULTS

### Respondent Characteristics

The pre-and post-intervention provider samples were mostly female (84% and 88%, respectively) and nurse-midwives (82% and 87%, respectively). While 65% of providers worked in the nursery or NBU pre-intervention, only 48% worked in the NBU post-intervention. The proportion of providers interviewed working in the postnatal/maternity and pediatric wards increased from pre- to post-intervention (20% to 28% and 15% to 24%, respectively) (Table 1).

**TABLE 1:** Characteristics of Providers Participating in Intervention to Improve Care for Hospitalized Young Children in Nairobi and Bungoma Counties, Kenya

Characteristic	Pre-Intervention, No. (%) (n=152)	Post-Intervention, No. (%) (n=103)	*P* Value
Providers who were female gender	128 (84.2)	90 (87.4)	.481
Providers working in hospital unit			
Postnatal/maternity ward	30 (19.7)	29 (28.2)	.020
Pediatric ward	23 (15.1)	25 (24.3)	
Nursery/newborn unit	99 (65.1)	49 (47.5)	
Provider type			
Doctor/clinical officer	27 (17.8)	14 (13.5)	.374
Nurse/midwife	125 (82.2)	90 (86.5)	
Age of providers, mean (SD), years (mean/SD)	38 (10.2)	39.5 (10.6)	.238

Abbreviation: SD, standard deviation.

The parent follow-up survey sample—which comprised all mothers of newborns and young children hospitalized in either the NBU or pediatric wards—were mostly aged 20–29 years (68%) and married (74%), with about half (50%) possessing a secondary education (Table 2). While 86% described newborn hospitalizations, most described their children recovering following hospitalization discharge (97%), with 2 reported deaths (1 from NBU and 1 from pediatric ward). On average, children were admitted for 8 days, with just over 8 days in the NBU and 4 days in pediatric wards. Most mothers (83%) said they were separated from their child during the hospitalization. The majority reported receiving some information on integrative responsive care during their child's hospitalization and showed moderately high levels of receipt of information (scoring 11 of 15).

**TABLE 2.** Characteristics of Mothers of Hospitalized Young Children Participating in Provider Behavior Change Intervention in Nairobi and Bungoma Counties, Kenya^a^

	Post-Intervention Only, No. (%)
Age of respondent, years	
15–19	27 (7)
20–24	145 (38)
25–29	116 (30)
30–34	53 (14)
>35	43 (11)
Level of education	
Primary and below	120 (31)
Secondary education	189 (50)
College	73 (19)
Marital status	
Single or divorced	101 (26.4)
Married	281 (73.6)
Child health status	
Child recovered/well	367 (97)
Baby still unwell	11 (3)
Baby died	2 (0.5)
Length of hospitalization, days	7.58 (8.39)
Child ever separated from parent	318 (83)
Ward	
Nursery/newborn unit	329 (86)
Pediatric ward	55 (14)
Received information on childcare through intervention, 0–15 index (SD)	11.6 (3.6)

Abbreviation: SD, standard deviation.

a Follow-up survey.

### Building Provider Knowledge and Capacity for Integrating Responsive Care

Implementation of the multifaceted intervention included tools (print and digital job aids) to support providers to enhance respectful and responsive care and communication strategies, as well as opportunities for providers to debrief on and strengthen their interactive partnership with parents. Post-intervention provider survey data demonstrated that of the 103 respondents, 44% of the providers used at least 1 job aid during their work. The most frequently used tools included the MOH clinical guide for child development (46%), the communication charter to guide provider-parent interactions developed by Breakthrough RESEARCH (41%), and a newly developed briefer on fathers' engagement in childcare (41%). Printed job aids placed in strategic areas of the facility made them easy to reference in practice. Respectful and responsive care-related videos in English and Swahili emerged as particularly popular in part because providers could access the videos at their convenience as well as conform to social distancing requirements during the COVID-19 pandemic. Seventy-five percent of providers watched at least 7 of 12 videos shared via WhatsApp by program staff. Providers reported most frequently watching videos related to skin-to-skin contact (55%), helping a breastfeeding mother (54%), and keeping a baby warm (49%). The least frequently watched videos were those on nonpharmacological measures for soothing pain (25%) and healing environments for preterm and sick infants (28%).

*With the videos, our work has become easier because now we have been displaying the videos and then we discuss the videos with the mothers on how to feed the babies and how to make locally available nutritious porridge.* —Nurse, NBU, post-intervention

While the provider orientation offered an initial opportunity to ask questions, most providers asked questions in the subsequent months when they met with nurse mentors, at monthly meetings, and via peer support. Provider survey data showed that close to 80% of providers found peer support on implementing the care components “very helpful.” Qualitative data further illustrated the value of monthly virtual meetings that provided a venue for interaction and sharing within and across different facilities, which enabled broader learning to occur.

*We interacted with other facilities. … We could share experiences and the video. We could see and it was very benefitting and educative.* —Nurse, mentor/deputy facility in-charge, post-intervention

Pre- and post-intervention provider surveys demonstrated some statistically significant increases in providers' knowledge around identification and addressing elements of integrative, respectful responsive care. Table 3 describes the pre- to post-implementation differences in summary index scores for each care element for newborns (aged 0–28 days) and young children (aged 29 days to 24 months). There were significant improvements in 3 of the elements for both age groups for safeguarding sleep, positioning and handling, and protecting skin. However, there were also significant reductions in providers' knowledge in identifying a child's pain, parental stress, and environmental stress. The only area where there was no change was optimizing nutrition, which started and remained at a higher level than most of the other elements.

**TABLE 3.** Knowledge Among Providers on Care of Hospitalized Young Children Pre- and Post-Intervention in Nairobi and Bungoma Counties, Kenya

Provider Knowledge	Pre-Intervention Score, Mean (SD) (n=152)	Post-Intervention Score, Mean (SD) (n=103)	*P* Value
Identifying child's pain and stress (0–7 items)	1.8 (1.2)	1.5 (1.3)	.011
Identifying parental stress (0–5 items)	2.5 (1.3)	1.8 (1.3)	<.001
Environment stress (0–5 items)	1.3 (1.1)	0.9 (1.1)	.001
Optimizing nutrition (0–14 items)^a^	4.8 (2.2)	4.9 (2.4)	.580
Safeguarding sleep (0–12 items)^a^	3.9 (1.9)	4.7 (2.4)	.004
Safeguarding sleep (0–12 items)^b^	3.9 (2.1)	5.1 (2.4)	.0003
Positioning/handling (0–8 items)^a^	2.3 (1.7)	3.9 (1.9)	<.001
Positioning/handling (0–8 items)^b^	1.8 (1.7)	4.0 (1.7)	<.001
Protecting skin (0–10 items)^a^	4.5 (2.2)	6.2 (2.1)	<.001
Protecting skin (0–7 items)^b^	3.2 (1.4)	4.5 (1.6)	<.001

Abbreviation: SD, standard deviation.

a Aged 0–28 days.

b Aged 29 days–24 months.

### Quality of Provider-Parent Communication

Pre- and post-intervention qualitative data showed improvement in provider behavior and communication within provider-parent interactions. Although some parents still found the attitudes of a few providers to be challenging, most parents found some improvement. A few parents described instances during their child's hospitalization pre- and post-intervention where they felt that they were “disturbing” a provider, and when parents asked for assistance or clarification, the provider responded, “Leave us [providers] to do our job.”

*I complained that I had found that the baby has removed the tube and I told the sister on duty my baby doesn't have a tube and she asked me where did it go, and I told her it is not there, and she said, what do you mean it is not there, where has it gone? You mothers have started removing the tubes from your babies; sometimes you really annoy us, we get tired. We will be giving you the tubes and then you put them on your babies yourselves.* —Woman, FGD, pre-intervention

*She was always on her phone. Even when you go to the nursery you find that the warmth is in excess and has made the kids cry and she does not look at how the meter is and yet she is there. If you ask her anything she responds arrogantly . . .* —Woman, IDI, post-intervention

Pre- and post-intervention qualitative data show improvement in provider behavior and communication within provider-parent interactions.

When providers appeared friendly, parents—both mothers and fathers—described being able to express themselves and ask questions post-intervention compared to pre-intervention.

*Some nurses are not friendly, what we call attitude and when you are attended to at a time like this when the life of your kid appears to be in the balance you need encouragement, attention, and assurance but now that one has not been forthcoming. You feel like you can even tell by the body language that this person feels like I am bothering him or her.* —Couple, IDI, pre-intervention

*When they came to check up on your baby and you asked how the baby was progressing, even if they were doing tests on the baby, they would stop to look at you and listen, then give you a response.* —Woman, IDI, pediatric ward, post-intervention

*It was also good because she was looking at me, checking me from time to time, checking the baby also and then coming to call me so that I go and check the baby because I used to forget to go and check the baby, this being my first child, so I had no experience.* —Woman, IDI, NBU, post-intervention

Some providers recognized there were challenges in how they communicated with parents of hospitalized children pre-intervention, but their perspectives concurred with parents around the enhanced quality of communication after implementation of the intervention.

*Providers can use abusive language like ‘you are untidy/unclean’ instead of counseling and training the mother, . . . . the affected mother stops to visit [come] again for services.* — Community health extension worker, IDI, pre-intervention

*For the relationship, if the provider and the mother are not in good terms, then there is a lot of information that this mother has about her baby that she is not going to disclose, which can help the provider in providing the [clinical]care [of the child].* —Nurse, IDI, pre-intervention

*Like if you are harsh to the parents, they may not be free to open up and tell you what the children are going through. There will be mistrust and misinformation that may misguide them in caring for their babies.* —Nurse, IDI, pre-intervention

Post-intervention, providers recognized the importance of communication.

*That [communication] makes the work a bit easier because parents interact freely and when it comes to counseling, … you have established rapport and when it comes to concern about the baby's condition it is easy because we are walking with the patient from day 1. …Everything that we do to the baby, we can explain to her and that has reduced so many questions that, “They are not telling me the service given to my baby.”* —Nurse, NBU, post-intervention

*It [intervention] has helped a lot. Like most of the cases we get in hospital … most of the problems come up because of communication. So, whenever you have good communication and handle the parents well, and all that, you never have a complaint in pediatric or an NBU.* —In-charge, NBU and pediatric ward, post-intervention

Quantitative data from the parents' follow-up survey reveal that parents rated the quality of communication with their providers as high, with some variability across several items within a composite measure (Table 4). The majority of parents rated very high on issues such as being spoken to gently (4%), using nonverbal gestures (95%), being listened to carefully (92%), clearly explained care (95%), and feeling confident to ask questions (92%). Almost all parents (99%) reported that the nurses answered their questions clearly using simple language.

**TABLE 4.** Proportion of Parents Rating Aspects of Provider's Communication Post-Intervention in Nairobi and Bungoma Counties, Kenya

Communication Aspect	Post-Intervention, No. (%) (N=382)
Greeted you in a friendly way	318 (83.2)
Introduced themselves	177 (46.3)
Explained any care that was required for your newborn/young child	334 (87.4)
Explained why tests were being carried out on your baby	278 (72.8)
Demonstrated and give examples while communicating	284 (74.3)
Gave mothers a chance to ask questions	324 (84.8)
You asked questions	311 (81.4)
Felt confident to ask questions about any aspect of your childcare	352 (92.1)
Answered your questions clearly using simple language based on responses from earlier question (N=311)	307 (98.7)
Explained all the examinations/procedures/tasks that s/he or you performed for your baby	285 (74.6)
Used a language you understand	366 (95.8)
Spoke gently to you and spouse/family	357 (93.5)
Listened carefully when you were talking/raising concerns about your child's care	352 (92.1)
Used nonverbal gestures to show you they cared (smile/laughter, eye contact, nodding)	364 (95.3)
Clearly explained to you about follow-up instructions for your baby's care	364 (95.3)
Told you what to do if you need to reach out to her for any concerns around your child	298 (78.0)
Interpersonal communication, overall score (0–16) (SD)	9.5 (6.4)

Abbreviation: SD, standard deviation.

In the follow-up survey, parents rated the quality of communication with their providers as high.

Qualitative data also show improvement in communication between pre-intervention and post-intervention, though there were still a few instances where parents and providers reported poor communication resulting in a negative experience of care.

*The one who was putting the line and the baby was pierced severally. I tried to express the pain of the baby, but the doctor was like “Leave me to do my work,” so he was harsh.* —Women, FGD, pre-intervention

*It [communication] has changed…. we have displayed the posters in our unit, and most of the time when we admit mothers, we tell them that these posters are in the wards, you can read them… Sometimes we review them as we [together] as we do the messages, so the decisions become easier… We listen to them, give them time, …[and] a chance to talk whatever they feel… The training taught us on how to relate with mothers.* —NBU provider, post-intervention

*Before the training [November 2020], Kangaroo Care was known. However, the nurturing care has not been active. But with training, we have been teaching the caregiver how to embody and communicate, sleep, and listen [to parents].* —Nurse mentor, NBU and pediatric ward, post-intervention

### Provider-Parent Coaching Empowering Parents in Integrative Responsive Care

**FIGURE 2:**
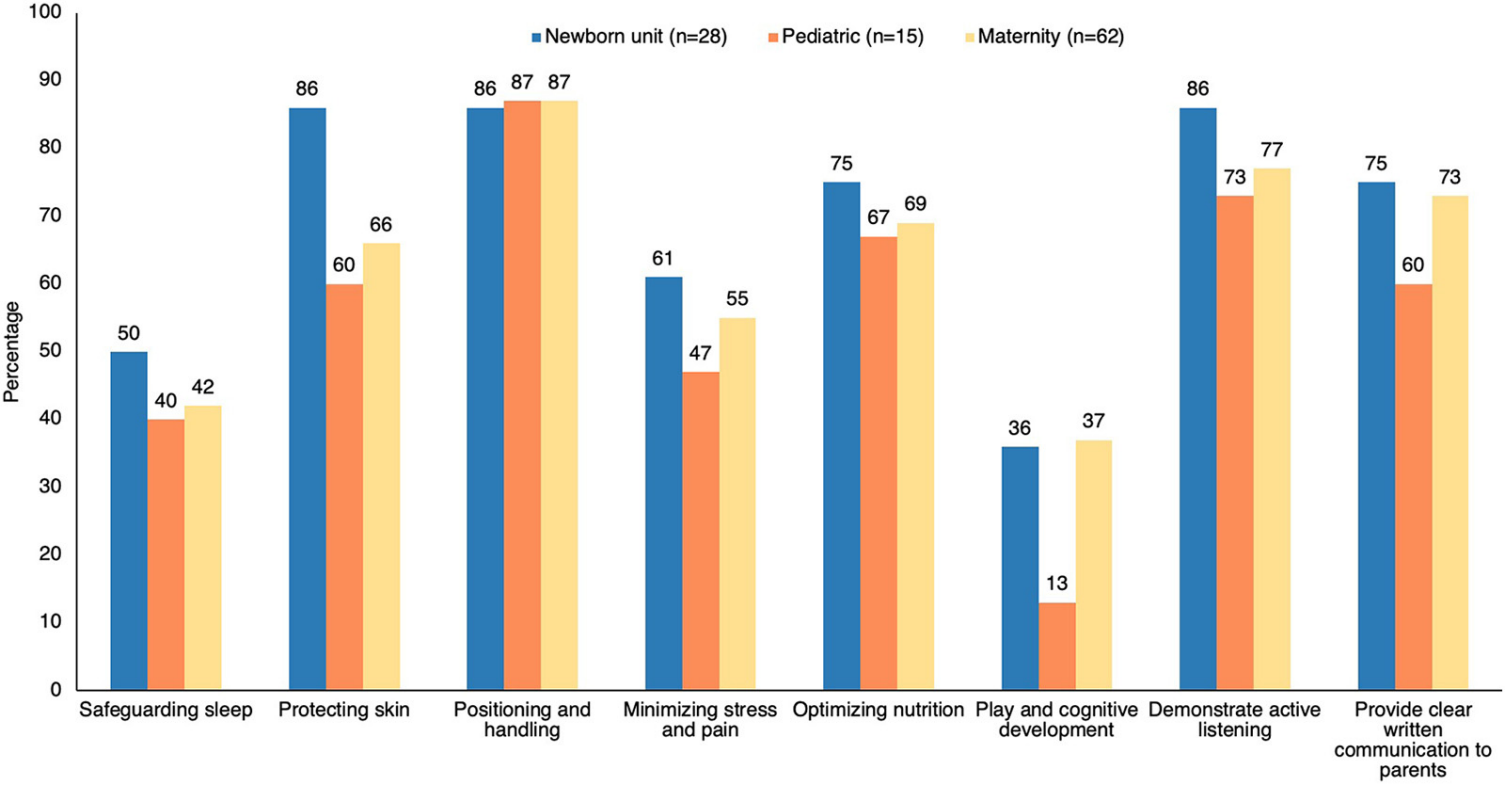
Providers Reporting Other Providers Involving Parents in Nurturing and Responsive Care Elements, Kenya Abbreviation: NBU, newborn unit.

During the intervention and post-intervention, parents and providers described moderate to high levels of interactive engagement around caring for hospitalized children alike. Monitoring data included provider-reported peer feedback using a short online questionnaire (see Methods section) and indicated that providers described varied but moderate levels of their peers' involving parents in elements of care over the last 6 months (Figure 2). The main reasons reported for nonengagement—particularly around safeguarding sleep, minimizing stress and pain, and promoting play and cognitive development—were inadequate time, low staffing, and limited space and supplies.

**FIGURE 3:**
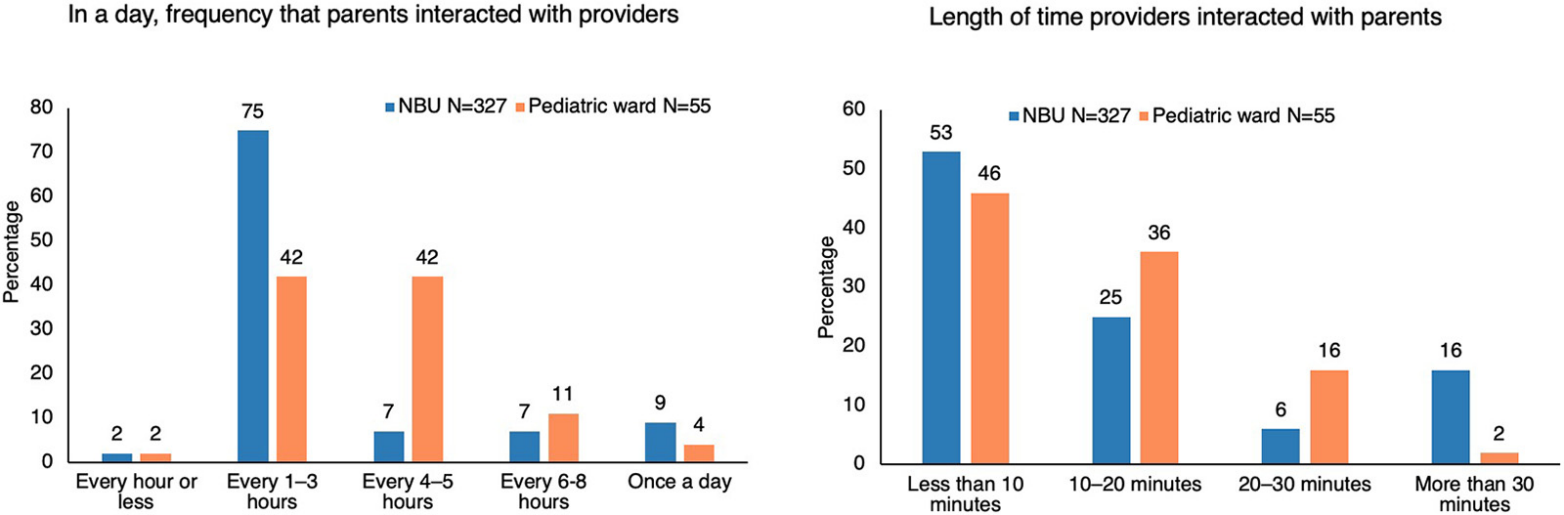
Frequency and Length of Provider-Parent Interactions During Young Children's Hospitalization in Kenya Abbreviation: NBU, newborn unit.

Most parents reported being counseled and coached by providers (90%), either individual (55%) or through group mentoring sessions, with some variability across wards. While 52% and 76% of parents reported receiving individual counseling with providers on care elements in NBUs and pediatric wards, respectively (*P*=.001), 76% and 24% reported receiving group mentoring (*P*=.000) in NBUs and pediatric wards, respectively (data not shown). Overall, 41% and 55% of parents reported individual conversations and in-group mentoring as the most helpful ways to receive information on their child's care. On average, parents mostly reported interacting with providers every 1–3 hours for less than 10 minutes per interaction (Figure 3). However, over a third of parents of newborns and half of parents of children in the pediatric ward reported that the providers spent 10–30 minutes interacting with them.

Moreover, many fathers desired to be more involved in their young child's care, as self-identified during the pre-intervention FGDs. There was some improvement at the endline in their engagement, particularly in settings where the hospital visiting hours were changed to accommodate when they were free to attend after work and enabled their inclusion.

*It made us friends and cultivated a good relationship. At the nursery there was a matron who was also very good.* —Father, pediatric ward, post-intervention

*… Because there is a policy of hospital…have fixed times for visiting. some fathers who stay far from their families, example work in Nairobi . . . ., and won't be allowed to see the child when they arrive. We had a management meeting to address it.* —Nurse, NBU, post-intervention

Parental follow-up surveys further show that 85% and 72% of mothers reported their spouses (fathers of the hospitalized newborn or young child) being able to visit freely during the newborn and young child's hospitalization, respectively (data tables not shown). While nearly two-thirds (63%) of mothers described that their spouses were able to get information about their hospitalized young child, less than 20% reported that fathers were able to actively discuss and voice preferences around their child's care with providers. Qualitative data show similar improvements in fathers' involvement, particularly in receiving coaching to support in childcare and providing emotional and financial support to mothers.

*When the baby was born, she had surgery done, the doctors gave us guidance on how to care for the babies and monitor them in case there is any other complications let them know early enough [timely]. They used to tell us what is to be done to the baby…they gave us directions being our firstborn when I had no idea of how to handle the baby… On discharge, they explained to us how we were to hold the baby and check if anything looks abnormal.* —Father, NBU, post-intervention

Providers' interactive coaching was seen through qualitative post-intervention interviews and quantitative data as helpful in enhancing parents' confidence in caring for their newborn/young child broadly and collaborating in nurturing care specifically.

*There is a way that it [coaching] has affected me because I did not know how to manage a young infant, there is nothing that I can tell you that I used to know but when I was coached in there, I was shown things, I can say that it has helped me a bit.* —Mother, NBU, post-intervention

Providers' interactive coaching was seen as helpful in enhancing parents' confidence in caring for their newborn/young child broadly and collaborating in nurturing care specifically.

Parent follow-up survey data present a generalized parent empowerment score that demonstrates over 90% of 382 parents reported confidence in performing tasks assigned by providers during hospitalization. Table 5 describes the item distributions and scores comprising the empowerment scale and ability to provide nurturing care composite index. Each of the 7 elements examined for empowerment had a score of 1–4, ranging from strongly disagree-1 to strongly agree-4, for a maximum score of 28 (Box 3). The 27 elements examined for nurturing and responsive care were split into 2 for young children aged 0–28 days, each with a score of between yes=1, sometimes=2, and no=0. A similar scoring system with 19 elements was used for children aged 29 days to 2 years.

**TABLE 5.** Mothers' Empowerment and Ability to Care for Their Young Child Post-Intervention in Nairobi and Bungoma Counties, Kenya

Elements Examined	Mothers With Children Hospitalized in Newborn Unit, No. (SD) (n=327)	Mothers With Children Hospitalized in Pediatric Ward, No. (SD) (n=55)	Overall Score, No. (SD) (n=382)	*P* Value
Empowerment score (0–28)	23.2 (3.08)	22.94 (2.92)	23.17 (3.05)	.543
Nurturing and responsive care elements				
Overall ability for parents to provide care for their newborn^a^ (0–27)	24.21 (2.69)	23.69 (2.14)	24.13 (2.62)	.172
Overall ability for parents to provide care for their young child^b^ (0–20)	18.72 (2.14)	18.54 (1.78)	18.69 (2.09)	.567

Abbreviation: SD, standard deviation.

a Aged 0–28 days.

b Aged 29 days–24 months.

BOX 3 Elements of Mothers' Empowerment and Ability to Care for Their Young Child

1. I feel in control of my newborn/young child's health.
2. I know what to do when my newborn/young child has a health problem.
3. I will be responsive to and care for my newborn/young child at home.
4. I can find a solution to my newborn/young child's health problem.
5. When my newborn/child is unwell, I advocate for them to get good care.
6. I can share information about caring for my newborn/child with my family/friends while in the hospital.
7. I can share information about caring for my newborn/child with my family/friends in my community.

### Associations Between Intervention Exposure and Parents' Self-Reported Outcomes

Implementation of the intervention yielded positive associations with parents' self-reported interpersonal communication with providers, parental empowerment for caring for their newborn/young child, and ability to care for their hospitalized child (Table 6). Controlling for age of parent, parental education, marital status, length of child's hospitalization, separation of mother/baby, and ward type, we found cross-sectional associations that showed differences between parents who reported receiving information on nurturing and responsive care compared to those who did not report receiving the same. Parents who received information during hospitalization on nurturing and responsive care also reported higher levels of interpersonal communication with providers (β=0.33; *P*=.000), empowerment in caring for their newborn/young child (β=0.28, *P*=.000), and self-reported ability to provide responsive nurturing care (β=0.14; *P*=.012).

**TABLE 6.** Associations Between Parents' Self-Reported Outcomes and Receipt of Integrative and Nurturing Care Information Post-Intervention in Nairobi and Bungoma Counties, Kenya^a^

Independent Variable	Higher Levels of Interpersonal Communication With Providers	Parental Empowerment to Care for Children	Ability to Provide Responsive Care to Child
Received care information through intervention	0.33^b^	0.28^b^	0.14^c^
Mother's age, years (ref=15–19)			
20–24	0.11	−0.61	1.24
25–29	0.25	−0.62	1.01
30–34	0.18	−0.17	1.74^c^
>35	0.56	0.13	1.47
Level of education (ref=primary and below)			
Secondary education	0.51	1.02^d^	−0.14
College	0.34	0.74	−0.01
Married/living together (ref= single/divorced)	0.18	0.87^c^	−0.06
Length of hospitalization, days	0.00	−0.01	0.04
Child ever separated from parent (ref=never)	0.72^c^	0.36^c^	0.93^c^
Pediatric ward (ref=newborn unit)	1.53^d^	1.15^c^	−0.95

a All results are adjusted beta coefficients controlling for the listed covariates in the first column.

b *P*<.000.

c *P*<.05.

d *P*<.001.

## DISCUSSION

We describe the feasibility of implementing a pilot PBC intervention to improve communication between providers and parents of hospitalized newborns and young children in Kenya, with transferable lessons for institutionalizing components in the long term. In developing the intervention, we drew from a range of frameworks and models,^1^^,^^4^ including the experience of care described in WHO quality of care frameworks for maternal and newborn health and child health comprising 3 domains: (1) effective communication and meaningful participation; (2) respect, preservation of dignity, protection, and fulfillment of child rights, and (3) emotional and psychological support.^2^^,^^3^

**FIGURE 4:**
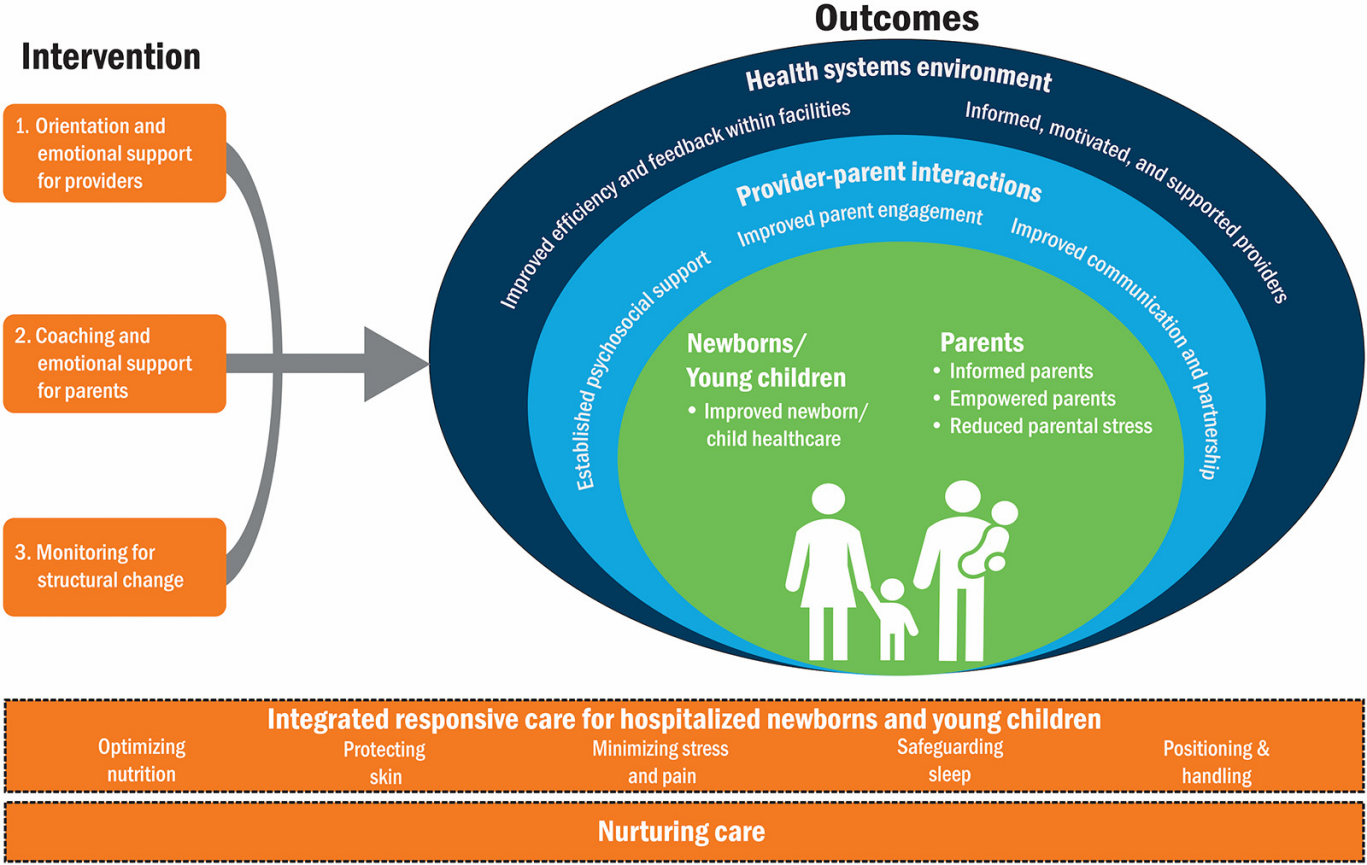
Theory of Change on Enhancing Respectful, Nurturing, and Responsive Care for Sick Young Children Aged 0–24 Months, Kenya^a^ ^a^ Parent, as described in the figure, denotes family caregiver, guardians, and extended family members.

We developed a theory of change during the codesign workshop (Figure 4), with 3 key areas of focus—orientation and emotional support for providers, coaching and emotional support for parents, and monitoring for structural change. We demonstrate that provider behavior rooted in these frameworks is a reasonable entry point to implement a range of pragmatic interventions in the Kenyan hospital setting and yield positive provider and parent experience. The implementation process influenced several outcomes including positive associations in improved providers' knowledge of respectful, responsive nurturing care, improved provider-parent interaction/communication, improved parental ability to provide responsive care to their babies, and parents' empowerment.

### Building Provider Knowledge and Capacity for Nurturing Care Is Critical

As demonstrated through national policy documents and guidelines, the Kenyan MOH is committed to scaling up WHO's frameworks for nurturing care, maternal and newborn health, and pediatric quality of care. However, as in many low-resource settings, often the quality of inpatient services for care of hospitalized newborns and very young children is limited by crowding, noise, lack of privacy, uncomfortable beds, lack of food and supplies, limited understanding of national policy at facility levels, inadequate and under-resourced health systems, and inadequate staff.^13^^,^^18^^,^^20^ Any intervention needs to be implemented as a multicomponent package anchored in a nurturing, integrative responsive care approach.^21^ Our study demonstrates that it is possible to build provider capacity and improve aspects of quality of newborn and young child care and communication with parents of hospitalized young children (i.e., the experience of quality of care as per the domains in the WHO maternal and newborn health and pediatric quality of care frameworks).

Although none of the mean scores reached the full number of items for aspect of care post-intervention, knowledge among providers increased significantly by endline, specifically around positioning and handling, protecting skin, and safeguarding sleep for both age groups (neonates aged 0–28 days and very young children aged 29 days to 24 months). There was no change around optimizing nutrition, which was high at baseline and remained high at endline, likely due to the influence of other implementing partners previously supporting the same facilities (e.g., UNICEF, PATH, and Save the Children) that have a specific focus on breastfeeding and infant feeding practices. Providers may also assume women would have received information on breastfeeding during antenatal clinics.

There were mixed results in provider understanding and identification of child pain and stress and parental stress. This is possibly due to normative views of pain in young children but also that stress and strain are not easy to measure and manage, especially when the provider is also under stress. Despite the provider training, qualitative data show that provider practices around managing pain and distress among hospitalized young children during the intervention period remained constrained. Although materials were supplied during training, the lack of national pain guidelines, supplies, and protocols at each facility resulted in some babies and infants not receiving any pain relief during procedures. This is a critical area that requires additional focus; there is potentially some normalization of pain in hospitalized young children in Kenya or at least insufficient motivation to do something about it, even though nonpharmacological ways to reduce pain and stress were introduced (and reinforced with a short video accessible to all providers) and can be readily adopted by parents and providers.^22^^,^^23^ This includes non-nutritive sucking, swaddling, and skin-to-skin contact 30 minutes before a painful procedure.^24^ Research shows respectful, responsive care is associated with improved newborn and young child health outcomes, provider and parent satisfaction, and a decrease in the number of days in hospital and costs^4^ and suggests integrability of nurturing and developmental care with child protection and parenting support interventions.^13^^,^^21^

Our qualitative and quantitative data demonstrated the overall positive effect of the intervention on provider-parent communication and partnership.

### Multistakeholder Perspectives on Quality of Provider-Parent Communication

Family-centered interventions target health care providers and parents of hospitalized young children and emphasize partnership in caring roles. Our qualitative and quantitative data demonstrated the overall positive effect of the intervention on provider-parent communication and partnership. A high proportion (over 90%) of parents described providers speaking gently, listening carefully, giving clear explanations on baby care, using a language a mother understood, using nonverbal gestures to show they cared, and the mother feeling confident to ask questions. Good communication between providers and parents is critical for building confidence and encouraging bonding and participation of mothers in the care of their babies. Other studies concur that family members report satisfaction with their health care experiences involving a family-centered approach due to the transparency of care and allowing parents to be at the infant's bedside.^25^ Family-centered care includes principles of dignity and respect, information sharing, participation, and collaboration, and it promotes a mutually beneficial partnership among parents, families, and health care providers to support health care planning, delivery, and evaluation.^9^^,^^13^ Our findings focused on parents of newborns and young children also align with person-centered maternity care frameworks that increasingly offer potential measures for monitoring experience of care from a client interacting with the health system.^3^

### Parental Empowerment in Caring for Their Hospitalized Newborn or Young Child

When parents are well informed, there is an increased likelihood of parental engagement and involvement in their child's care. They then feel part of any decisions made for their child's well-being.^26^^,^^27^ Boland et al. suggest that shared decision-making is not always implemented in a systematic way due to perceived lack of time by providers.^28^ Parents also report they receive insufficient quality information about their child's needs.^28^ Nevertheless, our study demonstrated high empowerment scores among parents (more than 90%) and confidence in performing tasks. More fathers felt more engaged in their child's care at endline as a result of removal of restrictive visiting hours (although not in all facilities) and being able to actively discuss and voice their preferences for their child's care with the providers. Overall, the more information parents received through higher levels of communication, the more they felt empowered and confident in caring for their hospitalized child.

The more information parents received through higher levels of communication, the more empowered and confident they felt in caring for their hospitalized child.

### Implications for Provider Behavior Change Programming on Caring for Hospitalized Newborns and Young Children

Focusing on provider behavior in a low-resource setting illuminated the importance of understanding providers' working environments and fostering an acceptable context before introducing any change. The results from the 2019 formative study were disseminated at 2 validation meetings with newborn and child health stakeholders—including program managers, policymakers, and advocates, as well as parents of previously hospitalized newborns and/or young children and health care providers—to validate the study findings and jointly develop/cocreate the study interventions. This resulted in a broader understanding of the specific hospital environment where providers, newborns and young children, and parents interact. The shared understanding—providers' understanding of parents' needs and parents' understanding of providers' working environment—resulted in a pragmatic behavior change intervention that built on several frameworks promoting responsive care of hospitalized newborns and young children. Providing a platform or model of care that everyone could relate to—with the young child at the center—enhanced communication across the board. A mix of mentoring (virtual and in person), physical job aids, soft-copy tools (videos and checklists), and monthly reflective discussions on monitoring data and feedback allowed for provider flexibility in using them to both learn and support communication with parents. Endline findings were shared through county and national newborn and child health technical working groups and a webinar led by Kenyatta National Hospital for over 150 clinicians, academicians, and members of professional organizations.

### Limitations

This study has some noteworthy limitations. The most challenging context was the COVID-19 pandemic, which led to several amendments to the design and data collection methods. Although we were able to hold an in-person codesign workshop to develop the overall intervention with parents and providers in early 2020, the study design shifted to phone-based data collection and virtual training of providers—both mentors and mentees. This prevented planned rounds of in-person data collection with parents and providers and observations of interactions between (1) providers and parents and (2) providers and young children. Specifically, some data/variables were only collected at 1 time point (such as the parent interviews post-intervention), which limited our ability to make causal inferences. The pandemic limited the length of intervention exposure, fidelity of the intended components, and our monitoring ability. We adapted intervention components, including planned in-person orientation and skills updates for providers, to remote activities, which restricted our implementation reach (of providers and parents). Another limitation is related to generalizability, given the relatively small sample size of parents and providers from the 5 hospitals that were interviewed over the phone. It is possible that we missed interviewing parents who did not have access to a phone or who live where cell phone signal is poor. Despite our limited ability to assess long-term sustainability and institutionalization, providers' use of job aids in their work and parents' willingness to provide experience of care feedback through short monitoring surveys that could be routinized suggest that PBC offers a promising strategy to affect the frequency and quality of communication interventions—including the application of a nurturing, integrative responsive care approach—to improve the care of hospitalized newborns and young children in low-resource settings like Kenya.

## CONCLUSION

Despite the challenges of implementing a PBC intervention to improve care for hospitalized newborns and young children during the global COVID-19 pandemic, we have demonstrated some promising results. It is feasible to implement a hybrid virtual and in-person process to influence several outcomes, including provider knowledge and practice, provider partnerships with parents, and parents' capacity to engage in the care of their newborn or young child. This approach can be used as an example for future studies and programs to embed a nurturing and integrative responsive care approach into similar settings in sub-Saharan Africa.

## Supplementary Material

GHSP-D-23-00004-supplement-2.pdf

GHSP-D-23-00004-supplement-1.pdf

## Notes

**Peer Reviewed**

**First published online:** July 27, 2023.
