# Supplementary material for: Lessons From a Behavior Change Intervention to Improve Provider-Parent Partnerships and Care for Hospitalized Newborns and Young Children in Kenya
Source: Glob Health Sci Pract. 2023 Nov 30;11(Suppl 1):e2300004. doi: 10.9745/GHSP-D-23-00004 (PMC10698236; doi:10.9745/GHSP-D-23-00004)
Supplement: GHSP-D-23-00004-supplement-2.pdf [file GHSP-D-23-00004-supplement-2.pdf]

**Supplement to**: Warren CE, Sripad P, Ndwiga C, et al. Enhancing a provider-parent experience: lessons from a provider behavior change intervention to improve care for hospitalized young children in Kenya. *Glob Health Sci Pract*. 2023;11(Suppl 3):e2300004. https://doi.org/10.9745/GHSP-D-23-00004

# Supplement 2

## Table S1: Items assessing providers on nurturing care elements (provider survey)

| | |
|---|---|
| **A** | **Minimizing stress and pain** |
| **#** | **Recognize pain and stress in a baby** - How do you tell that a baby is stressed or in pain (interviewer do not prompt, tick all that applies) |
| 1 | Sad |
| 2 | Limp and weak |
| 3 | Needle and tubes put on |
| 4 | Intravenous feeding or tube insertion |
| 5 | Jerky and restless |
| 6 | Bruises/cuts/ incisions |
| 7 | Can't cry |
| | **Overall score for child stress (0-7) (SD)** |
| | **How would you minimize pain in sick/young infant** |
| 1 | Use oral of paracetamol |
| 2 | Use glucose/sucrose in premature babies |
| 3 | Use of opioids e.g., morphine |
| 4 | Use of local anesthetics (topical application or injection) |
| 5 | Breastfeeding |
| 6 | Distraction e.g., music, playing with the child, showing bright colors |
| 7 | Applying heat or ice (warm/cold compress) on injection site /bruise |
| 8 | Gentle massage |
| 9 | Other specify |
| | **Overall score for child stress (0-9) (SD)** |
| | **How can you tell that the parents are stressed by the baby's illness?** |
| 1 | Cannot feed the baby |
| 2 | Cannot provide care to baby |
| 3 | Looks disinterested in baby |
| 4 | Exhibits restlessness /confusion/ agitation |
| 5 | Looks agitated |
| | **Overall score for parental stress (0-5) (SD)** |
| | **What environmental factors (what they see and hear) overwhelm parents in wards/facility?** |
| 1 | Noise from respirators |
| 2 | Sudden alarms/noise |
| 3 | Sight of monitors |
| 4 | Seeing many sick babies |
| 5 | Seeing many providers |
| | **Overall environmental stress (0-5) (SD)** |
| **B** | **Optimizing nutrition (0-28 days)** |
| | **How would you support a child up to 28 days to ensure adequate feeds and good feeding practices?** |
| 1 | Facilitate early and frequent exclusive breastfeeding |
| 2 | Encourage prolonged skin to skin contact |
| 3 | Promote positive oral/olfactory (smell) stimulation by letting the baby lick and smell the nipple |
| 4 | Ensure mother is competent with exclusive breast feeding before discharge |
| 5 | Counsel mothers on correct breastfeeding position |

| | |
|---|---|
| 6 | Provide space and chairs to ensure mothers comfort during breastfeeding |
| 7 | Encourage frequent breast feeding |
| 8 | Hold/ensure babies with NGT are well positioned |
| 9 | Support and encourage mothers to express milk for baby with NGT feeding |
| 10 | Ensure balanced diet for breastfeeding mothers |
| 11 | Ensure adequate feeding time for mother/baby at NBU |
| 12 | Use appropriate size NGT feeding |
| 13 | Coach and support mother on NGT feeding |
| 14 | Encourage a mother to have enough rest |
| | **Overall score optimism nutrition (0-14) (0-28 days)** |
| **C** | **Safeguarding sleep** |
| | **How would you support a newborn up to 28 days to safeguard sleep?** |
| 1 | Encourage prolonged skin-skin contact |
| 2 | Ensure and coach parent on nestled position for good sleep |
| 3 | Recognize and protect baby sleeping patterns |
| 4 | Provide flexible timing for care/procedures |
| 5 | Avoid interruptions/bright light |
| 6 | Arouse child using soft and gentle voice |
| 7 | Ensure adequate warm and right clothing |
| 8 | Encourage parents to observe child's sleep pattern and develop and sleeping routine |
| 9 | Coach, educate and mentor parents on the importance of sleep and rational for back -to -sleep and tummy- time |
| 10 | Protect baby's eyes from direct lighting |
| 11 | Avoid rough handling, noise or over stimulating the child |
| 12 | Ensure adequate feeds |
| | **Overall scores for safeguarding sleep (0-12) (SD) (0-28 days)** |
| | **How would you support a child between 29 days to 2 years to safeguard sleep?** |
| 1 | Ensure and coach parent on nestled position for good sleep |
| 2 | Recognize and protect baby/young child sleeping patterns |
| 3 | Provide flexible timing for care/procedures |
| 4 | Avoid interruptions/bright light |
| 5 | Arouse child using soft and gentle voice |
| 6 | Ensure adequate warm and right clothing |
| 7 | Encourage parents to observe child's sleep pattern and develop and sleeping routine |
| 8 | Coach, mentor parents on the importance of sleep and rational for back -to -sleep and different positioning |
| 9 | Protect baby's eyes from direct lighting |
| 10 | Avoid rough handling, noise or over stimulating the child |
| 11 | Ensure adequate feeds |
| | **Overall scores Safeguarding sleep (0-11) (SD) (29-2 yrs)** |
| **D** | **Positioning and handling** |
| | **How would you support correct positioning and handling for a newborn up to 28 days?** |
| 1 | Ensure skin- skin contact – where possible |
| 2 | Reposition child with care and minimally every four hours |
| 3 | Engage with child and let the behavior guide you (care for the infant and not to the infant) |
| 4 | Encourage, coach and mentor the parents on touching and cuddling the newborn |
| 5 | Encourage, coach and mentor the parents on developmentally appropriate stimulation and play as they mature |
| 6 | Collect/assemble all required items first |

| | |
|---|---|
| 7 | Involve the parent in procedures |
| 8 | Communication with child |
| | **Overall scores for positioning and handling (0-8) (SD)** |
| | **How would you support correct positioning and handling for a child between 29 days to 2 years?** |
| 1 | Ensure skin- skin contact – where possible |
| 2 | Reposition child with care and minimally every four hours |
| 3 | Engage with child and let the behavior guide you (care for the infant and not to the infant) |
| 4 | Encourage, coach and mentor the parents on touching and cuddling the newborn |
| 5 | Encourage, coach and mentor the parents on developmentally appropriate stimulation and play as they mature |
| 6 | Collect/assemble all required items first |
| 7 | Involve the parent in procedures |
| 8 | Communication with child |
| | **Overall scores for positioning and handling (0-8) (SD)** |
| **E** | **Protecting skin (0-28 days)** |
| | **How would you support protecting skin for a newborn?** |
| 1 | Drying and wrapping newborn immediately after birth |
| 2 | Early, frequent, and prolonged skin to skin contact |
| 3 | Keep the room/areas of care warm and humid (e.g. NBU, labor ward, Postal ward, Pediatric ward and OPD) |
| 4 | Ensure NBU is clean during mothers feeding time |
| 5 | Use only water for bathing the child |
| 6 | Avoid harsh soap, lotions and detergent for bathing and washing the baby/cloths |
| 7 | Frequent nappy change |
| 8 | Wrapping and clothing the baby (not too warm) |
| 9 | Minimize adhesives use and pricking to find IV site |
| 10 | Teach the mother on how to provide developmentally appropriate infant massage to promote relaxation, bonding, and attachment |
| | **Overall scores for protecting skin (0-10) (SD) (0-28 days)** |
| | **How would you support protecting skin for a day 29-2 years?** |
| 1 | Frequent prolonged skin to skin contact as necessary |
| 2 | Keep the room/areas of care warm and humid (e.g. NBU, labor ward, Postal ward, Pediatric ward and OPD) |
| 3 | Avoid harsh soap, lotions and detergent for bathing and washing the baby/cloths |
| 4 | Frequent nappy change |
| 5 | Wrapping and clothing the baby (not too warm) |
| 6 | Minimize adhesives use and pricking to find IV site |
| 7 | Teach the mother on how to provide developmentally appropriate infant massage to promote relaxation, bonding, and attachment |
| | **Overall scores for protecting skin (0-7) (SD) (29 days-2 years)** |

**Supplement to**: Warren CE, Sripad P, Ndwiga C, et al. Enhancing a provider-parent experience: lessons from a provider behavior change intervention to improve care for hospitalized young children in Kenya. *Glob Health Sci Pract*. 2023;11(Suppl 3):e2300004. https://doi.org/10.9745/GHSP-D-23-00004

## Table S2: Items assessing parents experience of the interventions (parents' follow-up survey)

| # | **Did you receive information on?** |
|---|---|
| 1 | Exclusively breastfeed your newborn/infant or feed your young child |
| 2 | Keep your baby warm |
| 3 | Change diapers |
| 4 | Maintain personal hygiene |
| 5 | Bathe and keep your baby clean |
| 6 | Gently touching or soothing the baby |
| 7 | Talking to your baby/child |
| 8 | Allow pricking by providers when necessary |
| 9 | How to insert an NG tube (at hospital) |
| 10 | Nest (wrap/position) the baby |
| 11 | Skin to skin in the facility and at home |
| 12 | Maintaining a quiet environment when baby/child is sleeping |
| 13 | Mother baby breastfeeding positions |
| 14 | Changing a baby's position (not laying or sitting in one position for too long) |
| 15 | Stimulate/play with your baby/young child |
| | **Average score for information given (0-15)** |

**Supplement to**: Warren CE, Sripad P, Ndwiga C, et al. Enhancing a provider-parent experience: lessons from a provider behavior change intervention to improve care for hospitalized young children in Kenya. *Glob Health Sci Pract*. 2023;11(Suppl 3):e2300004. https://doi.org/10.9745/GHSP-D-23-00004

## Table S3: Items assessing quality of interpersonal communication (parent's follow-up survey)

| # | **During hospital stay …** |
|---|---|
| 1 | Did the provider greet you in a friendly way? |
| 2 | Did the provider introduce her/himself? |
| 3 | Did the provider explain any care that was required for your newborn/young child? |
| 4 | Did the provider explain why tests were being carried out on your baby? |
| 5 | Did the provider demonstrate and give examples while communicating? |
| 6 | Did the provider give you a chance to ask questions? |
| 7 | Did you ask any questions? |
| 8 | Did you feel confident to ask questions about any aspect of your childcare? |
| 9 | Did the provider answer your questions clearly using simple (non-medical) language? |
| 10 | Did the provider explain to you all the, examinations/ procedures/ cares tasks that s/he or you performed for your baby? |
| 11 | Did the provider use a language you understand (local language)? |
| 12 | Did the provider speak gently to you and spouse/family? |
| 13 | Did providers listen carefully when you were talking/ raising concerns about your childcare |
| 14 | Did providers use non-verbal gestures to show you they cared? E.g. smile, eye contact, nodding, |
| 15 | Did the provider clearly explain to you about follow up instructions for your baby's care? *By this I mean drugs, care task to be taken at home including the return date* |
| 16 | Did the provider tell you what to do in case you need to reach out to him/her for any concerns on care of your child? |
|  | **Average score for information given (0-16)** |

**Supplement to**: Warren CE, Sripad P, Ndwiga C, et al. Enhancing a provider-parent experience: lessons from a provider behavior change intervention to improve care for hospitalized young children in Kenya. *Glob Health Sci Pract*. 2023;11(Suppl 3):e2300004. https://doi.org/10.9745/GHSP-D-23-00004

**Table S4: Items assessing parent's empowerment to care for their child**

| Section 4- Parent empowerment | | | |
|---|---|---|---|
| 401. | I feel in control of my newborn/young child's health. | Strongly disagree……………………………………………1<br>Disagree…………………………………………………………2<br>Agree…………………………………………………3<br>Strongly Agree………………………………………4 | |
| 402. | I know what to do when my newborn/young child has a health problem. | Strongly disagree……………………………………………1<br>Disagree…………………………………………………………2<br>Agree…………………………………………………3<br>Strongly Agree………………………………………4 | |
| 403. | I will be responsive to and care for my newborn/young child at home. | Strongly disagree……………………………………………1<br>Disagree…………………………………………………………2<br>Agree…………………………………………………3<br>Strongly Agree………………………………………4 | |
| 404. | I can find a solution to my newborn/young child's health problem. | Strongly disagree……………………………………………1<br>Disagree…………………………………………………………2<br>Agree…………………………………………………3<br>Strongly Agree………………………………………4 | |
| 405. | When my newborn/young child is unwell, I advocate for them to get good care. | Strongly disagree……………………………………………1<br>Disagree…………………………………………………………2<br>Agree…………………………………………………3<br>Strongly Agree………………………………………4 | |
| 406. | I can share information about caring for my newborn/young child with my family/friends while in the hospital. | Strongly disagree……………………………………………1<br>Disagree…………………………………………………………2<br>Agree…………………………………………………3<br>Strongly Agree………………………………………4 | |
| 407. | I can share information about caring for my newborn/young child with my family/friends in the community. | Strongly disagree…………………………………1<br>Disagree……………………………………………………2<br>Agree…………………………………………………3<br>Strongly Agree………………………………………4 | |

**Supplement to**: Warren CE, Sripad P, Ndwiga C, et al. Enhancing a provider-parent experience: lessons from a provider behavior change intervention to improve care for hospitalized young children in Kenya. *Glob Health Sci Pract*. 2023;11(Suppl 3):e2300004. https://doi.org/10.9745/GHSP-D-23-00004

## Table S5: Items assessing parent's knowledge and ability to provide nurturing and responsive care

| **A** | **Nurturing and responsive care for newborns** |
|---|---|
| 1 | I know how to wrap my newborn safely so he/she can sleep |
| 2 | I never wake a sleeping baby unless necessary |
| 3 | I talk in a "soft voice" when near bedsides |
| 4 | I make sure direct light doesn't shine on my sleeping newborn |
| 5 | I know how to do skin to skin with my newborn at the hospital / home |
| 6 | A newborn needs a quiet environment to sleep |
| 7 | Newborns need to dress warmly at all times |
| 8 | My newborn is always kept in a warm room |
| 9 | Newborns diapers need to be changed regularly to keep them dry |
| 10 | I check my newborn's mouth for oral thrush and diaper area for rash regularly |
| 11 | I know how to keep the cord clean and dry |
| 12 | I used chlorohexidine (CHX) for umbilical cord care on my newborn (0-28 days) |
| 13 | I wash my hands with soap and water before I hold or care for my newborn |
| 14 | I wash my hands with soap and water (or wipe my breast) every time I feed my newborn |
| 15 | I know how to position my newborn to breastfeed |
| 16 | I change my newborn's position regularly |
| 17 | I know how to nest/wrap my newborn. |
| 18 | Pricking my newborn numerous times causes pain and stress |
| 19 | I know how to gently insert the naso-gastric tube for feeding |
| 20 | When holding a newborn, you need to use slow gentle movements |
| 21 | I know how to soothe my newborn |
| 22 | I talk to my newborn often. |
| 23 | A newborn need to be breastfed within one hour of birth |
| 24 | Newborns need to be fed the first breastmilk (colostrum) for a strong immune system and to prevent jaundice |
| 25 | Exclusive breastfeeding on demand is good for my newborn |
| 26 | Breast milk contain important anti-bodies and nutrients for my newborn |
| 27 | Mothers need a balanced diet to ensure they can provide adequate breast milk |
| **B** | **Nurturing and responsive care for older children 29 days – 2 years** |
| 1 | I never wake a sleeping child unless necessary. |
| 2 | I talk in a "soft voice" when near bedsides |
| 3 | I make sure direct light doesn't shine on my sleeping child |
| 4 | I know how to do skin to skin with my child at the hospital / home |
| 5 | A child needs a quiet environment to sleep |
| 6 | A young child needs to be dressed warmly at all times |
| 7 | My child is always kept in a warm room |
| 8 | I change my child's diapers regularly |
| 9 | I wash my hands with soap and water when I care for my child |
| 10 | I wash my hands with soap and water (or wipe my breast) every time I feed my child |
| 11 | My baby needs to be stimulated regularly for their health and development |
| 12 | Pricking my child numerous times causes pain and stress |
| 13 | When holding a child, you need to use slow gentle movements |

| | |
|---|---|
| 14 | I know how to soothe my child when they are in distress |
| 15 | I talk to my child often. |
| 16 | I play with my child often. |
| 17 | If baby is ≤6 months, I (or my spouse) am/is exclusively breastfeeding |
| 18 | If baby is ≤6 months, I/my spouse are not giving any liquids, not even water. |
| 19 | If baby is ≥ 7 months of age, I/my spouse have been giving complimentary food. |
| 20 | If baby is on complimentary feeds, are you able to provide a health balance diet during the feeds |
