# Supplementary material for: Lessons From a Behavior Change Intervention to Improve Provider-Parent Partnerships and Care for Hospitalized Newborns and Young Children in Kenya
Source: Glob Health Sci Pract. 2023 Nov 30;11(Suppl 1):e2300004. doi: 10.9745/GHSP-D-23-00004 (PMC10698236; doi:10.9745/GHSP-D-23-00004)
Supplement: GHSP-D-23-00004-supplement-1.pdf [file GHSP-D-23-00004-supplement-1.pdf]

COMMUNICATION DURING HOSPITALIZATION

HEALTHCARE PROVIDERS		PARENTS/ CAREGIVERS
Introduce yourself and your role<br>Be respectful, polite, and empathetic<br>Use local language and speak slowly	**ADMISSION**	Introduce yourself and your child<br>Be respectful, polite, and patient
Explain and decide on the child's care plan with parents/caregivers and provider team<br>Provide regular updates to parents and family promptly<br>Listen carefully to parents'/caregivers' questions and concerns<br>Answer any questions or concerns raised by parents/caregivers or refer appropriately	**CARE PLAN**	Ask for information about your hospitalized child's care plan<br>Voice your opinion about your child's care plan<br>Raise questions about your child's care<br>Ask for updates on your child's treatment plan
Allow and actively engage parent/caregiver participation in ward rounds<br>Explain clinical results and child's progress to parents/caregivers during ward rounds	**WARD ROUNDS**	Ask for an explanation of your child's progress during ward rounds<br>Seek information on test results and next steps in care
Explain the feeding methods for sick children, why, and how often to feed their child<br>Counsel the mother on breastfeeding and coach her on how to express breast milk if child cannot suckle<br>Show parents/caregivers how to feed their child and how to use weight charts	**FEEDING**	If your child is less than 6 months old exclusively breastfeed your child and ask how to breastfeed or express breast milk<br>If your child is more than 6 months old, feed your child a balanced diet and breastfeed on demand<br>If your child is unable to eat by mouth, ask to participate in feeding your child<br>Assist in filling information on your child feeding and weight charts
Explain why the NGT procedure is needed and ally any concerns<br>Explain how the procedure will be done and ensure the child's pain and/or discomfort is minimized<br>Explain how to care for the child after the NGT insertion<br>Demonstrate feeding methods so parents can support their child (including care of the tubes)	**INSERTION OF NASAL GASTRIC TUBES (NGT)**	Support the provider during tube insertion by soothing, comforting, and talking to your child<br>Support the provider in caring for the tubes and follow infection prevention rules<br>Ask if you can participate in NGT feeding and for the provider to demonstrate NGT feeding<br>Note: your child's feeding method may change from time to time depending on your child's condition
Inform the parents/caregivers about the procedure, why it is required, and what is involved<br>Allow the parents/caregiver to be present and encourage them to help comfort the infant if feasible<br>**MAXIMUM** 3 attempts allowed then you must seek help (**use discretion with very small infants or infants with difficult access)**	**INTRAVENOUS (IV) CANNULAS FOR TREATMENT**	Use comfort measures as needed (swaddling, non-nutritive suckling, warmth, shield child's eyes from direct lighting)<br>Help the provider check for signs of inflammation (swelling, pain, coolness of skin, and leakage at site)
Orient the parents/caregivers on the ward environment, use of bathrooms, and sleeping areas for parents/caregivers<br>Share information on the use of feeding spaces and incubators<br>Explain infection prevention control measures on hand washing and wearing masks<br>Explain facility policies on visitation and family members<br>Ensure a calm and soothing environment for sleep by minimising bright lights and loud noises<br>Coach the caregiver on how to reduce pain and discomfort by breastfeeding, swaddling, cuddling, singing/humming, suckling for soothing, and skin-to-skin care when in the hospital and to practice upon discharge	**WARD ENVIRONMENT** (INCLUDING FEEDING, SLEEPING)	Ask about the ward environment and visitation policies<br>Seek information for your comfort and safety, as well as warmth and safety of your child such as cots, rails, and warmers<br>Seek information on how to support sleep and minimise your child's pain during and after procedures<br>Follow provider's advice on how to breastfeed, swaddle, sing or hum, suckle for soothing, and skin-to-skin care<br>Inform providers when child shows signs of distress<br>Provide feedback to the provider on experience of care (complete the exit forms using phone, SMS, or suggestion box)

USAID FROM THE AMERICAN PEOPLE
POPULATION COUNCIL Ideas. Evidence. Impact.
Breakthrough RESEARCH FOR SOCIAL & BEHAVIOR CHANGE

PARENTS' EMOTIONAL WELLNESS:

Reduce Distress, Emotional Support, and Partnership (DEP) Guide for Supporting Parents During a Young Child's Illness

Will help in identifying sources of anxiety, fears, and concerns, providing emotional support, and engaging with parents and families to mitigate stress.

DISTRESS

D

Questions to ask parents to assess distress in families

Assess newborn's/young infant's pain.
- ✓ How do you think your child is doing right now?
- ✓ How is the child's pain right now?
- ✓ What was the worst thing that happened since you came to the hospital?

Gauge fears and worries.
- ✓ What has been scary or upsetting for you?
- ✓ What worries you the most?

Address grief or loss.
- ✓ Was anyone else hurt or ill?
- ✓ Have you had other recent losses (home, family member)?

Tips to help reduce distress in families

1. **Provide parent with as much control as possible.**
   - Help the parent understand what is happening.
   - Allow the parent to have a say in what will happen next.
2. **Actively assess and treat the child's pain.**
   - Minimize pain and exposure to bright lights/loud sounds.
   - Teach parent basic coping techniques, e.g., breastfeeding, non-nutritive suckling, actively distracting, swaddling, and singing to the child during procedures.
3. **Provide accurate information, using basic words.**
   - Ask the parent to repeat back explanations.
   - Listen carefully and clarify misconceptions.
4. **Provide reassurance, realistic hope, and comfort with dignity.**
   - Describe what is being done to help the child feel better.
   - Address the parent's concerns or worries.
   - Find a quiet, private place to talk and project warmth, interest, and respect.

EMOTIONAL SUPPORT

E

Questions to ask parents to assess the family's emotional needs

Assess child's current needs.
- ✓ What helps you or your child cope when upset/scared?

Identify who will be able to support the child.
- ✓ Do you understand the illness/injury or treatment?
- ✓ Can you be with your child during procedures?
- ✓ Can you help calm/soothe their child?

Identify barriers to mobilizing parent support.
- ✓ How confident are you in caring for the child?
- ✓ Think to yourself: Do any of the parents' responses make it harder for them to help?
- ✓ What would make it easier for you to help care for the child?

Tips to help provide emotional support to families

1. **Listen to parents and encourage their presence.**
   - Ask parents for their expertise about their child.
   - Ask parents about their concerns.
   - Encourage them to be with their child.
2. **Empower parents to help their child.**
   - Suggest ways they can help their child—soothing/comforting, ACTIVE distraction before and during procedures.
   - Involve them in physical/clinical care, as appropriate.
   - Help them seek out support if upset/anxious.
3. **Encourage child/parent involvement in "normal" activities.**
   - Suggest activities that the child and parent can do together, e.g., reading, singing, story telling.

PARTNERSHIP

P

Questions to ask parents to assess how family is coping (including beyond medical)

Assess distress of family members.
- ✓ How is your family coping right now?
- ✓ Who is having an especially difficult time?
- ✓ What does your family think about it?

Gauge family stressors and resources.
- ✓ Are you eating, getting sleep, and taking breaks?
- ✓ Do you have friends who can help out at home?

Address other needs (beyond medical).
- ✓ Are there other stressors going on (such as money, job, transportation) that make it particularly difficult right now?

Tips to help families cope with bereavement for the current medical situation and other issues that are impacting them

1. **Encourage parents' basic self-care.**
   - Encourage parents to sleep, eat, and take breaks.
   - Help them enlist support of friends, family, and community.
2. **Remember other family members' needs.**
   - Involve siblings and explain treatment to them when possible.
   - Enlist hospital resources such as chaplain and social work as needed.
3. **Be sensitive to the cultural and resource needs of the family.**
   - Remember that outside issues can impact recovery.
   - Connect families with community resources they trust.
   - Show respect by working with the family.

Adapted from Healthcare Toolbox

USAID FROM THE AMERICAN PEOPLE

POPULATION COUNCIL Ideas. Evidence. Impact.

Breakthrough RESEARCH FOR SOCIAL & BEHAVIOR CHANGE

PROVIDERS' EMOTIONAL WELLNESS

A, B, Cs of provider self-care when working with sick children and their families.

Awareness

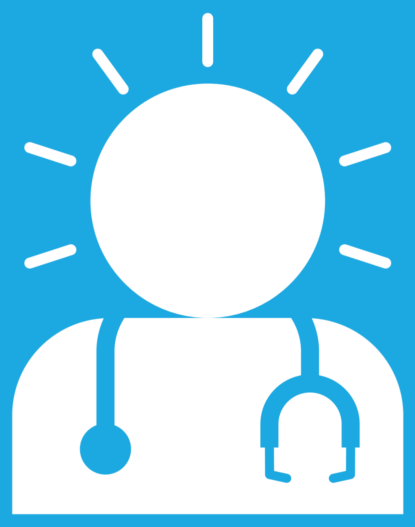

- ✓ Be aware of how you react to stress (overworking, overeating, exhaustion, and verbal outbursts).
- ✓ Monitor your stressors and set limits with parents and colleagues.
- ✓ Talk to a professional or your manager if your stress affects your life or relationships.

Balance

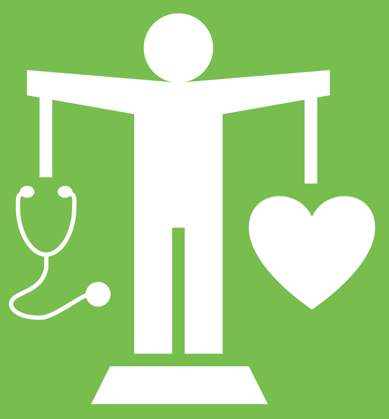

- ✓ Diversify tasks and take breaks during the workday.
- ✓ Eat sensibly, exercise regularly, and get enough sleep.
- ✓ Engage in activities outside of work; use your leave days.

Connection

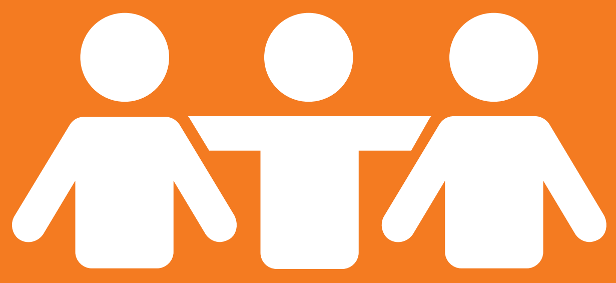

- ✓ Connect regularly with family, friends, and community.
- ✓ Use meditation, prayer, or relaxation to connect with yourself.
- ✓ When not at work, disconnect from professional role and e-mail and/or WhatsApp messages.

Adapted from Saakvitne & Pearlman, 1996

USAID FROM THE AMERICAN PEOPLE
HARAMBEE
POPULATION COUNCIL Ideas. Evidence. Impact.
Breakthrough RESEARCH FOR SOCIAL & BEHAVIOR CHANGE

WHAT CAN MEN DO?

Fathers and other male carers can do the following for newborns and young children

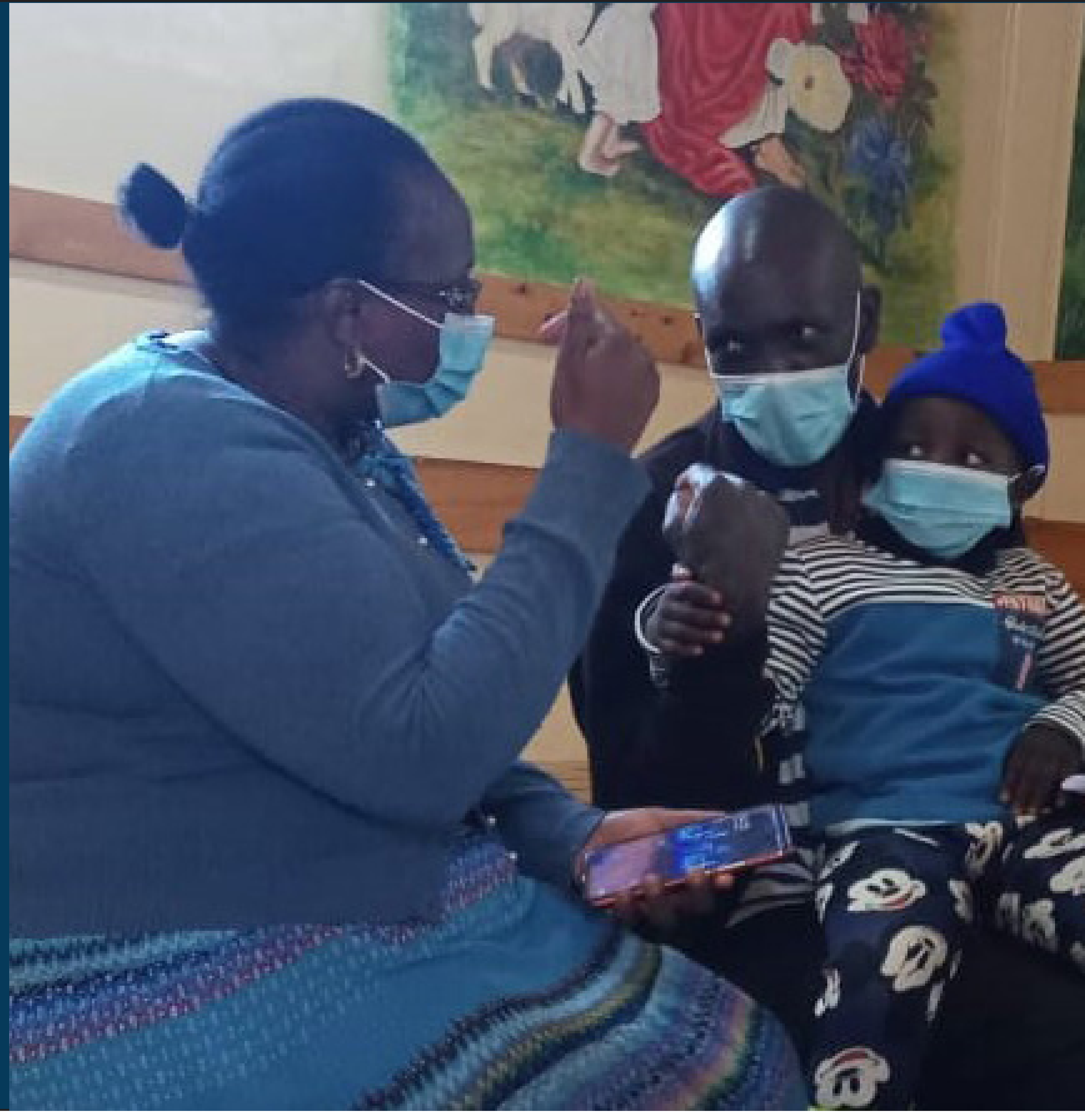

WASH HANDS WITH SOAP AND WATER

Before touching the baby

Before and after feeding the baby

Before and after preparing food

Before and after changing nappies

After using the toilet

PARTICIPATE IN FEEDING

Encourage and support mother during breastfeeding

Feed the baby with a cup or spoon

Provide age-appropriate foods for the baby

Help prepare the food

Ensure mother has nutritious foods and liquids for herself during breastfeeding

SKIN-TO-SKIN CONTACT

Place baby on your chest as often as possible for warmth, to reduce stress, and to strengthen father-baby bonding

DECISION MAKING

While child is sick promptly seek care to prevent complications and follow-up on treatment recommendations

Seek information on child's progress during care

Participate in healthcare decisions and discussions

PLAY WITH AND SOOTHE YOUR BABY

Talk and sing to your baby

Hold your baby before, during, and after procedures to minimize pain and stress

STAY ENGAGED AND INFORMED

Speak to providers directly if you have any questions or concerns

Engage in family discussions to determine emotional and financial needs for the child

USAID FROM THE AMERICAN PEOPLE

HARAMBEE

POPULATION COUNCIL Ideas. Evidence. Impact.

Breakthrough RESEARCH FOR SOCIAL & BEHAVIOR CHANGE

SOURCE | HTTPS://WWW.FAMILYINITIATIVE.ORG.UK/NEONATAL-FATHERS
